# Siglec-G Deficiency Ameliorates Hyper-Inflammation and Immune Collapse in Sepsis via Regulating Src Activation

**DOI:** 10.3389/fimmu.2019.02575

**Published:** 2019-11-07

**Authors:** Wenqian Li, Yinjiao Li, Kewei Qin, Boxiang Du, Tianliang Li, Hongbin Yuan, Chaofeng Han, Yan Luo

**Affiliations:** ^1^Department of Anesthesiology, Changzheng Hospital, Second Military Medical University, Shanghai, China; ^2^Department of Anesthesiology, Ruijin Hospital, Shanghai Jiaotong University School of Medicine, Shanghai, China; ^3^Central Laboratory, The Sixth Medical Center of Chinese PLA General Hospital, Beijing, China; ^4^The Second Affiliated Hospital of Nantong University, Nantong, China; ^5^National Key Laboratory of Medical Immunology, Institute of Immunology, Second Military Medical University, Shanghai, China

**Keywords:** Siglec-G, Src homology region 2 domain-containing phosphatase-1, Src, sepsis, pro-inflammatory cytokines, anti-inflammatory cytokine

## Abstract

Hyper-inflammation during acute phase and sequential hypo-inflammation during immunosuppressive phase in macrophages/monocytes lead to multiorgan failure syndrome and immune collapse of sepsis, in which toll-like receptor (TLR)-triggered inflammatory responses play a major role. Here, we reported that *Siglecg* deficiency attenuated TLR4-triggered pro-inflammatory cytokine production and increased anti-inflammatory cytokine [interleukin-10 [IL-10]] production *in vivo* and *in vitro* at both acute and immunosuppressive phases. *Siglecg* deficiency also protected mice from lipopolysaccharide (LPS)-induced sepsis with less inflammation in the lung and less tissue destruction in the spleen. Siglec-G inhibited proto-oncogene tyrosine-protein kinase Src (Src) activation via recruiting and activating tyrosine phosphatase Src homology region 2 domain-containing phosphatase-1 (SHP1) through immunoreceptor tyrosine-based inhibitory motif (ITIM) domain. Src could inhibit TLR4-induced inflammatory cytokines and promote anti-inflammatory cytokine IL-10. Mechanical investigation showed that Src could interact with and phosphorylate STAT3. Src could also promote HIF1α degradation through activating GSK3β. Our study reveals that Siglec-G orchestrates TLR-induced inflammation, which outlines that blocking Siglec-G or activating Src may be a promising strategy for both acute and chronic inflammatory diseases.

## Introduction

Sepsis is a systemic inflammatory syndrome induced by infection, caused by the lack of normal immune homeostatic functions and excessive production of pro-inflammatory cytokines, which leads to multiorgan failure and immune collapse (1–7). Toll-like receptor (TLR) signaling and inflammatory responses are critical in the pathology of sepsis. By recognizing pathogen-associated molecular patterns (PAMPs), TLRs initiate innate immune responses by activating signaling pathways that depend on the adaptor myeloid differentiation primary response 88 (MyD88) or TIR-domain-containing adapter-inducing interferon-β (TRIF) and consequently induce the production of pro-inflammatory cytokines and type I interferon (IFN) (8–10). The positive and negative regulation of TLR signaling has been under intensive research (9, 10). Diverse signaling molecules have been identified to be essential for full activation of TLR responses (9, 10). However, negative regulators of TLR such as Src (11) can prevent over-activation of TLR signaling, which may result in inflammatory disorders or autoimmune diseases (12, 13). We previously found that CD11b-activated Src signaling could inhibit inflammatory cytokine in sepsis (11) and could promote anti-inflammatory cytokine IL-10 in mice model of colitis (14). Whether, there is a signal pathway regulating both inflammatory and anti-inflammatory cytokines in acute inflammation remains unknown (9, 15). Searching the balance point of pattern recognition receptor (PRR) signal transduction to produce appropriate production of anti-inflammatory and pro-inflammatory cytokines is a key question for innate responses and a promising drug target (9, 15).

Siglecs are divided into two groups on the basis of their molecular structure. The first group includes Siglec-1, Siglec-2, Siglec-4, and Siglec-15, which are structurally conserved between rodents, humans, and other vertebrates (16–19). The second group includes Siglec-3/CD33 and CD33-related Siglecs, which are less structurally conserved between human and other vertebrates but highly homologous to CD33 in their extracellular domains. Most Siglecs bind sialic acid ligands either in-cis (on the same cell) or in-trans (on a neighboring cell or on a microbe) and provide inhibitory signals to immune cells. Siglecs play an important role in cell signal transduction, pathogen recognition, phagocytosis, and treatment of tumors. Siglec-15 could induce immunosuppressive tumor microenvironment via immunoreceptor tyrosine-based activation motif (ITAM) domain of DAP12 (20). Our lab first cloned Siglec-10 from human dendritic cells (DCs) (21), which is a member of the CD33-related Siglec family in the mouse. Recent studies demonstrated that Siglec-G had a board negative modulation on B cells (17). Siglec-G also inhibited DC cross-presentation by impairing major histocompatibility complex (MHC)-I peptide complex formation (22). In innate immunity, inflammatory responses to host danger-associated molecular patterns (DAMPs) are repressed by the interaction of CD24 and Siglec-G (23, 24). We previously reported that Siglec-G negatively regulated RNA virus-induced type I IFN production by promoting c-Cbl-mediated ubiquitination and degradation of retinoic acid-inducible gene 1 (RIG-I) via SHP2 (25). Most Siglecs have ITIM or ITIM-like motifs in intracellular domains. Upon ligand recognition, Siglecs recruit tyrosine phosphatases such as SHP1 or SHP2 to regulate signal transduction through ITIM or ITIM-like domain (22, 25, 26). Furthermore, Siglec-G deficiency decreased infiltration of inflammatory cells and inflammation in fat tissue (27), which is consistent with the negative function of ITAM in inflammation (12, 28). However, the role of ITIM-containing Siglec-G and the function of SHP1/2 in acute inflammation remain unclear.

In this study, we observed that *Siglecg* deficiency protected mice from over-activation of acute inflammatory responses and death in TLR-triggered sepsis by attenuating TLR-triggered pro-inflammatory cytokine production and increasing anti-inflammatory cytokine IL-10 production. We further demonstrated that Siglec-G decreased Src activation through SHP1. Our results showed that Siglec-G-induced Src signaling could be a promising drug target to regulate immune homeostasis of pro-inflammation and anti-inflammation.

## Results

### Siglec-G Orchestrates Toll-Like Receptor-Triggered Inflammatory and Anti-inflammatory Cytokine Productions *in vivo*

**Figure 1:**
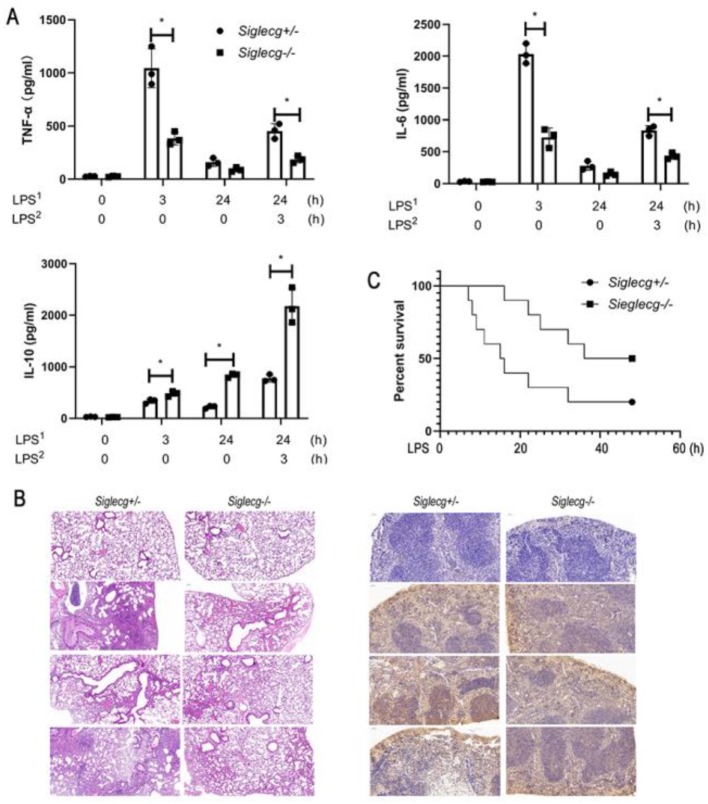
*Siglecg*-deficient mice are more resistant to LPS-induced sepsis. **(A)** Enzyme-linked immunosorbent assay (ELISA) of TNF-α, IL-6, and IL-10 in serum from *Siglecg*^+/−^ and *Siglecg*^−/−^ mice injected intraperitoneally with PBS or LPS (5 mg/kg) as indicated. Data are representative of three independent experiments with similar results and presented as means ± SD. ^*^P < 0.01. **(B)** Hematoxylin and eosin staining of the lungs (left panel); the immunostaining of phosphor-p65 (middle panel) in the spleen from *Siglecg*^+/−^ and *Siglec*^−/−^ mice after intraperitoneal injection of PBS or LPS as indicated. Original magnification: ×10 for the lungs and ×20 for the spleen. Data are representative of three independent experiments with similar results. **(C)** Survival of *Siglecg*^+/−^ and *Siglecg*^−/−^ mice (*n* = 10 per genotype), monitored every hour after challenge with lethal dose of LPS (15 mg/kg). *P* < 0.01 (Wilcoxon test). LPS, lipopolysaccharide; IL, interleukin; TNF-α, tumor necrosis factor-α; PBS, phosphate-buffered saline.

We first found that the baseline expression of Siglec-G was not the highest, but its expression was upregulated significantly upon lipopolysaccharide (LPS) stimulation (Supplementary Figure 1). To investigate the role of Siglec-G in the inflammatory responses of sepsis, we challenged *Siglecg*-deficient (*Siglecg*^−/−^) and control (*Siglecg*^+/−^) mice with a non-lethal dose of LPS for indicated time, a sepsis model we previously reported (29). In response to the first LPS challenge, *Siglecg*^−/−^ mice produced less interleukin-6 (IL-6) and tumor necrosis factor-α (TNF-α) than did *Siglecg*^+/−^ mice (Figure 1A). However, *Siglecg*^−/−^ mice produced more anti-inflammatory cytokine IL-10 than did *Siglecg*^+/−^ mice. Twenty-four hours after the first LPS challenge, the second LPS challenge induced significant lower production of IL-6 and TNF-α, which implicated an immunosuppressive status. *Siglecg* deficiency also significantly decreased the second LPS-induced pro-inflammatory cytokine (IL-6 and TNF-α) productions, whereas it increased the anti-inflammatory cytokine IL-10 production compared with that in the control mice. We observed more severe infiltration of inflammatory cells in the lungs of *Siglecg*^+/−^ mice than that in the lungs of *Siglecg*^−/−^ mice at both acute and immunosuppressive phases (Figure 1B). Accordingly, there was less activation of NF-κB (phosphorylation of p65 subunit) and tissue destruction in the spleen of *Siglecg*^−/−^ mice than in *Siglecg*^+/−^ mice. Upon lethal LPS-dose challenge, 60% of *Siglecg*^+/−^ mice remained alive for 16 h, but only 20% of them ultimately survived, whereas 80% of *Siglecg*^−/−^ mice ultimately survived (Figure 1C), which indicated that *Siglecg*^−/−^ mice were significantly more resistant to LPS-induced sepsis than were control mice. These results suggest that *Siglecg* deficiency protects mice from sepsis in both acute and immunosuppressive phases by orchestrating TLR-triggered inflammatory responses by inhibiting NF-κB activation.

### Siglec-G Orchestrates Toll-Like Receptor-Triggered Inflammatory and Anti-inflammatory Cytokine Productions in Macrophages

**Figure 2:**
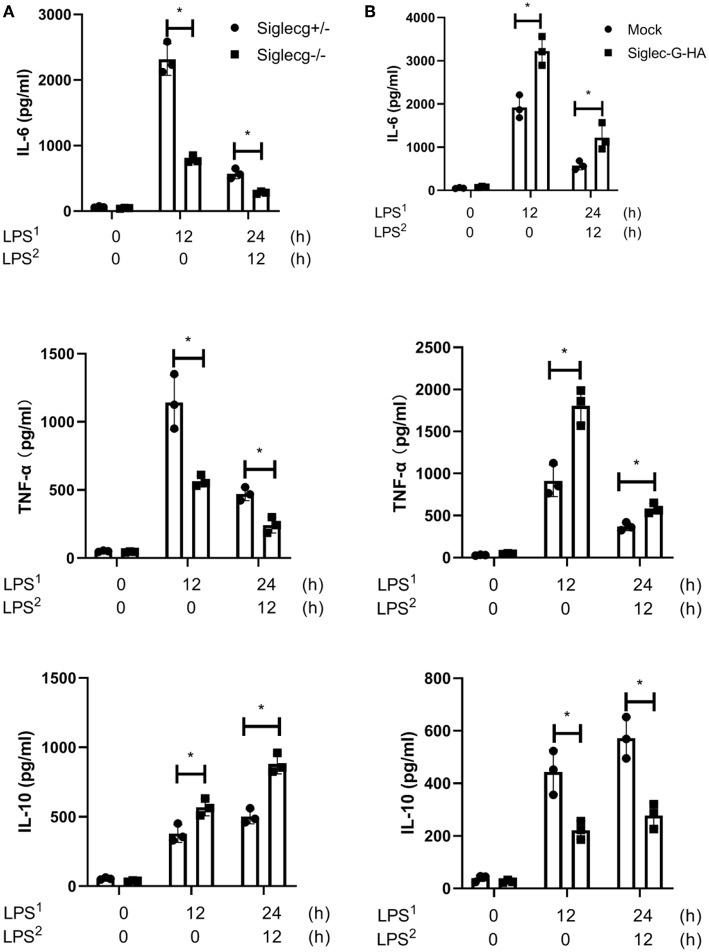
Siglec-G orchestrates TLR-triggered pro-inflammatory and anti-inflammatory cytokines production in macrophage. (**A**) ELISA of IL-6, TNF-α, and IL-10 in supernatant from *Siglecg*^+/−^ and *Siglecg*^−/−^ peritoneal macrophages (6 × 10^5^) that were stimulated with first round of LPS (100 ng/ml) as indicated or were changed with then fresh medium for the second round of LPS for 12 h. **(B)** ELISA of IL-6, TNF-α, and IL-10 in supernatant from Siglec-G-overexpressing RAW264.7 macrophages (8 × 10^5^) stimulated with LPS as **(A)**. Data are representative of three independent experiments with similar results and presented as means ± SD. ^*^P < 0.01. TLR, toll-like receptor; IL, interleukin; TNF-α, tumor necrosis factor-α; LPS, lipopolysaccharide.

**Figure 3:**
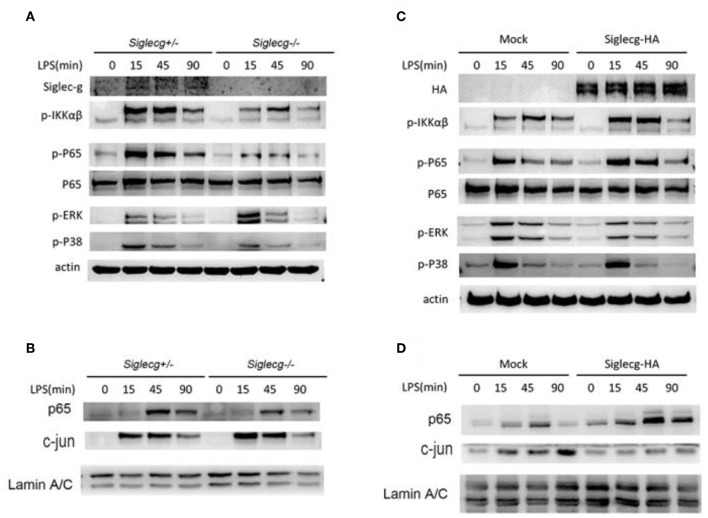
Siglec-G orchestrates NF-κB and ERK activation. **(A,B)** Immunoblotting of cell lysates **(A)** or nuclear extract **(B)** from *Siglecg*^+/−^ and *Siglecg*^−/−^ 2 × 10^6^ peritoneal macrophages stimulated with LPS (100 ng/ml) for the indicated time with indicated antibodies. **(C,D)** Immunoblotting of cell lysates **(C)** or nuclear extract **(D)** from Siglec-G overexpressing RAW264.7 cells (3 × 10^6^) stimulated with LPS (100 ng/ml) for the indicated time with indicated antibodies. Data are representative of three independent experiments with similar results. ERK, extracellular signal-regulated kinase; LPS, lipopolysaccharide.

The above data indicated that Siglec-G orchestrated TLR-triggered inflammatory and anti-inflammatory cytokine productions *in vivo*. To further confirm the function of Siglec-G *in vitro*, LPS-induced cytokine production in mouse peritoneal macrophages was tested using ELISA. In response to LPS administration, *Siglecg*^−/−^ peritoneal macrophages produced less pro-inflammatory cytokines (IL-6 and TNF-α) but more IL-10 than did *Siglecg*^+/−^ (Figure 2A, Supplementary Figure 2) and S*iglecg*^+/+^ (Supplementary Figure 2) macrophages at both acute and immunosuppressive phases. The mRNA expression of pro-inflammatory cytokines also decreased, and the mRNA expression of IL-10 increased in (*Siglecg*^−/−^) macrophages (Supplementary Figure 3). Then we further investigated whether overexpression of Siglec-G could increase pro-inflammatory cytokines production in RAW264.7 cells. Overexpression of Siglec-G-HA significantly increased LPS-induced IL-6 and TNF-α production and decreased IL-10 production than in control cells at both acute and immunosuppressive phases (Figure 2B). The knockout and overexpression efficiency was verified in the next part (Figures 3A,C). These data further confirmed that Siglec-G could promote TLR-trigged pro-inflammatory cytokine production and inhibit anti-inflammatory cytokine IL-10 production *in vitro*.

### Siglec-G Orchestrates NF-κB and Extracellular Signal-Regulated Kinase Activation

To further explore how Siglec-G promotes TLR-induced pro-inflammatory cytokine production, the downstream signal pathway was tested. The expression of Siglec-G in macrophages and knockout efficiency were first verified. We found that the activation of NF-κB was impaired in *Siglecg*^−/−^ macrophages compared with that in *Siglecg*^+/−^ macrophages (Figure 3A), which was evidenced by decreased phosphorylated IKKα/β and p65 levels. However, the activation of extracellular signal-regulated kinase (ERK), which is critical for TLR-induced IL-10 production (30), was increased in *Siglecg*^−/−^ macrophages. The nuclear translocation of NF-κB transcriptor p65 was decreased, and the ERK-induced transcriptor AP-1 (c-jun) was increased in *Siglecg*^−/−^ macrophages than in *Siglecg*^+/−^ macrophages (Figure 3B). The Siglec-G was overexpressed efficiently in RAW264.7 cells. We also tested whether *Siglec-G* overexpression could orchestrate the activation of NF-κB and ERK in RAW264.7 cells. In accordance with results of *Siglecg*-deficient macrophages, the activation of NF-κB was also increased in Siglec-G-HA overexpressed RAW264.7 cells (Figure 3C). And Siglec-G-HA overexpression decreased the activation of ERK in RAW264.7 cells. Moreover, similar results were confirmed by the nuclear translocation of p65 and AP-1 (c-jun) in Siglec-G overexpressed cells (Figure 3D). The above data suggested that Siglec-G could orchestrate TLR-induced NF-κB and ERK activation.

### Siglec-G Orchestrates Toll-Like Receptor Signaling Through Src

**Figure 4:**
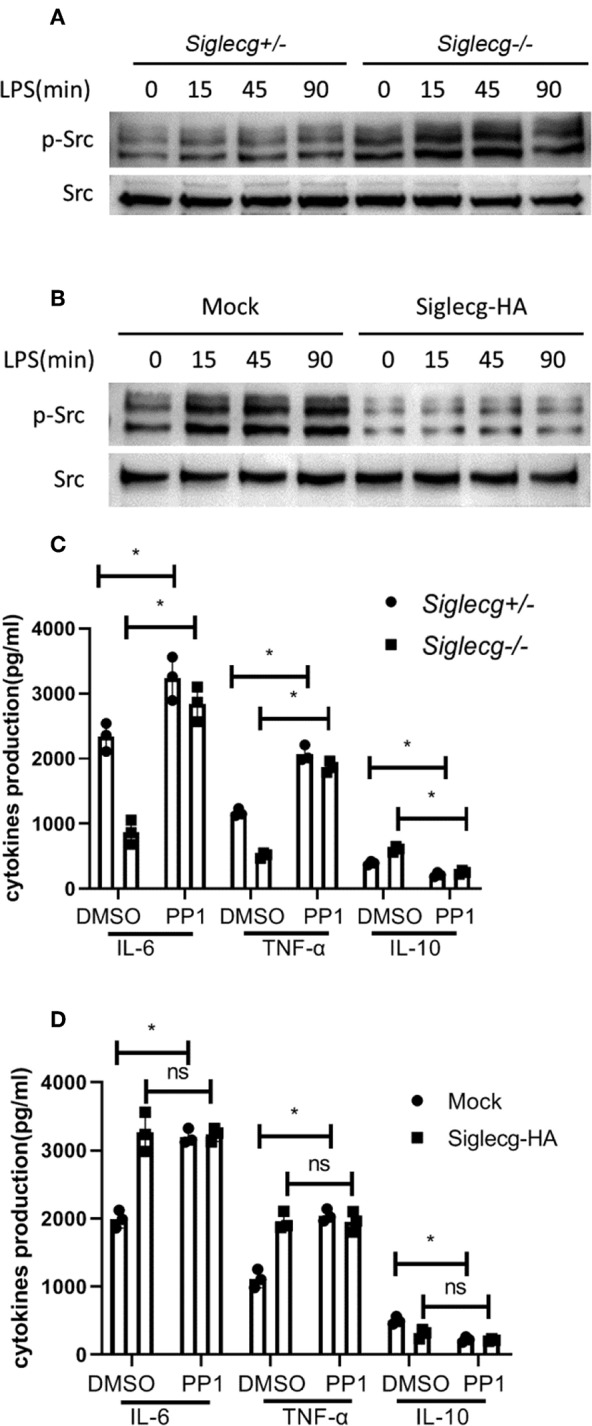
Siglec-G inhibits Src activation. **(A,B)** Immunoblotting of cell lysates from 2 × 10^6^ *Siglecg*^+/−^ and *Siglecg*^−/−^ peritoneal macrophages **(A)** or 3 × 10^6^ Siglec-G overexpressing RAW264.7 cells **(B)** stimulated with LPS (100 ng/ml) for the indicated time with indicated antibodies. Data are representative of three independent experiments with similar results. **(C,D)** ELISA of IL-6, TNF-α, and IL-10 in supernatant from *Siglecg*^+/−^ and *Siglecg*^−/−^ peritoneal macrophages **(C)** or Siglec-G overexpressing RAW264.7 macrophages **(D)** pretreated with PP1 (5 μM) for 2 h and then stimulated with LPS (100 ng/ml) for 12 h. Data are representative of three independent experiments with similar results and presented as means ± SD (ns, no significant differences; ^*^P < 0.01). LPS, lipopolysaccharide; IL, interleukin; TNF-α, tumor necrosis factor-α.

We previously found that CD11b-induced Src activation was involved in TLR signaling (8, 9). The activation of Src was increased in *Siglecg*^−/−^ peritoneal macrophages upon LPS stimulation (Figure 4A). And Siglec-G overexpression impaired Src activation (Figure 4B). Pretreatment of wild-type (WT) peritoneal macrophages and *Siglecg*^−/−^ peritoneal macrophages with Src inhibitor PP1 increased pro-inflammatory cytokine (IL-6 and TNF-α) production (Figure 4C) at both the protein and mRNA levels (Supplementary Figure 4). Accordingly, PP1 also decreased IL-10 expression. PP1 increased pro-inflammatory cytokines production more efficiently in *Siglecg*-deficient macrophages (Figure 4C), in which there was more strength of Src activation (Figure 4A). To further confirm the role of Src in TLR signaling, we pretreated Siglec-G overexpressed or control RAW264.7 cells with PP1. PP1 also increased IL-6 and TNF-α production and decreased IL-10 production in control RAW264.7 cells (Figure 4D), but not in Siglec-G overexpressed cells, in which the activation of Src was inhibited (Figure 4B). The above data indicated that Siglec-G regulated NF-κB and ERK activation through Src.

### Siglec-G Inhibits Src Activation via Src Homology Region 2 Domain-Containing Phosphatase-1

**Figure 5:**
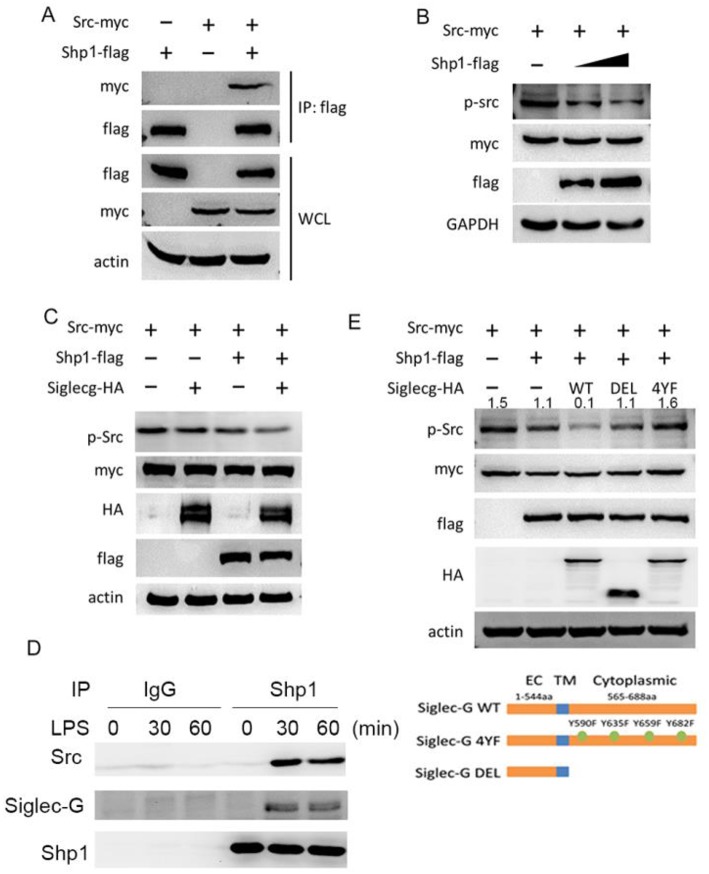
Siglec-G inhibits of Src activation via SHP1. **(A)** Immunoblotting of immunoprecipitated production or cell lysates from HEK293T cells overexpressing indicated plasmids. **(B,C)** Immunoblotting of the cell lysates from HEK293T cells overexpressing indicated plasmids. **(D)** Immunoblotting of immunoprecipitated production from macrophages with indicated antibodies. **(E)** Immunoblotting of the cell lysates from HEK293T cells overexpressing indicated plasmids. Data are representative of three independent experiments with similar results. SHP1, Src homology region 2 domain-containing phosphatase-1.

We further investigated how Siglec-G inhibited Src activation upon LPS stimulation. As we previously noticed a positive function of the tyrosine phosphatase SHP1 in innate immunity (28, 31), we investigated whether the positive function of Siglec-G was dependent on SHP1. Overexpression and co-IP experiments in HEK293T cells indicated that Src interacted with SHP1 (Figure 5A). Furthermore, we found that SHP1 could dephosphorylate overexpressed Src in a dose-dependent manner (Figure 5B). Overexpression of Siglec-G, SHP1, and Src in HEK293T cells indicated that Siglec-G could promote dephosphorylation of Src, which was further enhanced by SHP1 (Figure 5C). SHP1 could also interact with Siglec-G and Src upon LPS stimulation in macrophages (Figure 5D). Siglec-G contains an intracellular tail with four tyrosine-based motifs, one or two belonging to the ITIM domain. The Siglec-G ITIM inactive mutant (Siglec-G-4YF) decreased the inhibitory function on Src activation compared with the full-length Siglec-G (WT), whereas the cytoplasmic domain-deleted Siglec-G (DEL) mutant totally lost the inhibitory function on Src activation (Figure 5E) in the overexpressed system. These results indicated that Siglec-G could decrease Src activation by recruiting SHP1.

### Siglec-G Orchestrates STAT3 and HIF1α Activation via Src

**Figure 6:**
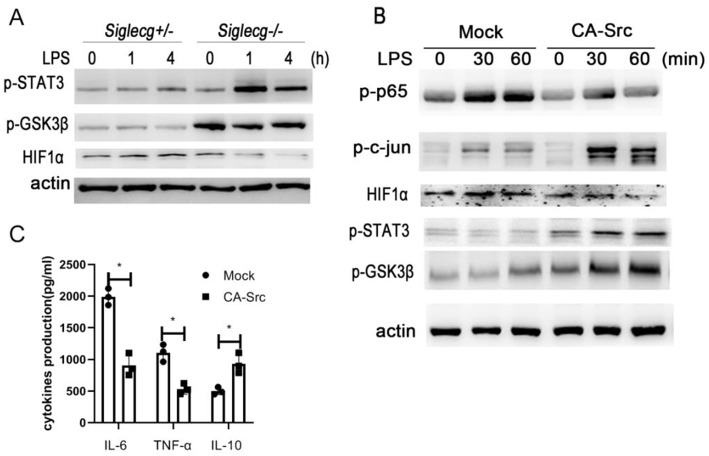
Siglec-G orchestrates TLR4-induced STAT3 and HIF1α activation via Src. **(A,B)** Immunoblotting of cell lysates from *Siglecg*^+/−^ and *Siglecg*^−/−^ peritoneal macrophages or RAW264.7 cells overexpressing CA-Src stimulated with LPS (100 ng/ml) for indicated time with indicated antibodies. **(C)** ELISA of IL-6, TNF-α, and IL-10 in supernatant from constitutively active Src (CA-Src) overexpressing RAW264.7 cells stimulated with LPS (100 ng/ml) for 12 h. Data are representative of three independent experiments with similar results as means ± SD. ^*^P < 0.01. TLR, toll-like receptor; LPS, lipopolysaccharide; IL, interleukin; TNF-α, tumor necrosis factor-α.

We investigated how Src orchestrated the inflammatory signaling activation. HIF1α is important in LPS-induced inflammatory response (32). We found that *Siglecg*^−/−^ macrophages exhibited lower HIF1α protein expression than *Siglecg*^+/−^ macrophages upon LPS stimulation (Figure 6A). GSK3β could phosphorylate HIF1α and promote its degradation in proteasome. Accordingly, the activation of GSK3β was increased in *Siglecg*^−/−^ macrophages compared with *Siglecg*^+/−^ macrophages. Furthermore, the activation of STAT3 is critical for IL-10 production (30, 33). Its activation increased in *Siglecg*^−/−^ macrophages compared with *Siglecg*^+/−^ macrophages upon LPS stimulation. We previously found that Src-activated Src- Akt-GSK3 signaling could activate AP-1 (14). Then we further investigated how Siglec-G-mediated Src signaling could increase AP-1 and STAT3 activation and IL-10 production. The activation of inflammatory signaling of NF-κB and HIF1α was decreased, while activation of anti-inflammatory signaling of AP-1, STAT3, and GSK3β was increased in RAW264.7 cells overexpressed constitutively active Src (CA-Src) (Figure 6B). Accordingly, RAW264.7 cells that overexpressed CA-Src also produced less IL-6, while they produced more IL-10 (Figure 6C). These data indicated that Src might promote HIF1α degradation and STAT3 activation to orchestrate inflammatory responses.

### Src Is Involved in STAT3 and HIF1α Activation

**Figure 7:**
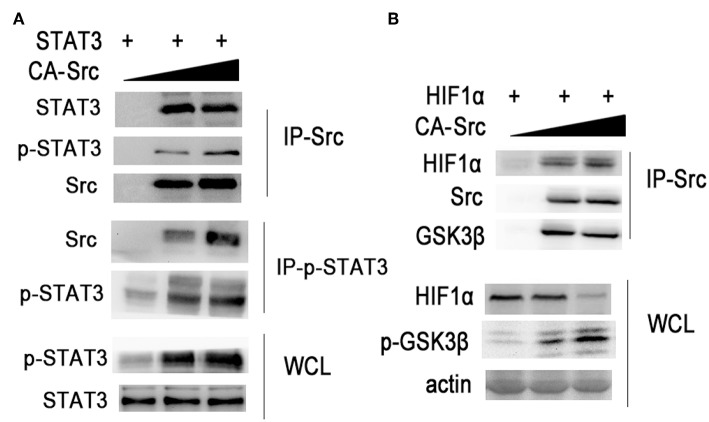
Src regulates STAT3 and HIF1α activation. **(A,B)** Immunoblotting of immunoprecipitated production or cell lysates from HEK293T cells overexpressing indicated plasmids with indicated antibodies. Data are representative of three independent experiments with similar results.

Then we further investigated the role of Src in regulating STAT3 and HIF1α activation. We co-overexpressed STAT3 with increased amounts of CA-Src in HEK293 cells (Figure 7A). Overexpressed CA-Src could interact with STAT3 and phosphorylated STAT3. CA-Src could increase the phosphorylation with STAT3 in a dose-dependent manner. When HIF1α was co-overexpressed with increased amounts of CA-Src in HEK293 cells, CA-Src could also interact with GSK3β and HIF1α (Figure 7B). CA-Src could also increase the phosphorylation of GSK3β and decrease the expression HIF1α in a dose-dependent manner. The above results show that Src was involved in the inflammatory signaling by regulating STAT3 and HIF1α activation.

## Discussion

Sepsis is defined as a life-threatening organ dysfunction with systemic mixed pro-inflammatory and anti-inflammatory responses to an infectious organism or severe tissue injury (1, 6, 7, 15, 34). Despite the failure of numerous clinical trials, anti-inflammatory treatment strategies in the early hours after the onset of sepsis are still a promising option (3). Indeed, clinical studies showed that subgroups of patients with sepsis might benefit from anti-inflammatory treatment strategies such as IL-1 receptor blockade or anti-TNF treatments (6, 7, 34). Here, we found that Siglec-G deficiency orchestrated the pro-inflammatory cytokines and anti-inflammatory cytokine IL-10, which protected mice from LPS-induced sepsis. Interestingly, applying inhibition of Siglec-G downstream Src or activating Src could significantly orchestrate the pro-inflammatory cytokine and anti-inflammatory productions in macrophages. Although anti-inflammatory cytokines blocking therapy will cause protracted immunosuppressive phase in most patients (5–7, 15, 34), our results indicated that blocking Siglec-G (activating Src) in hyper-inflammation phase or activating Siglec-G (inhibiting Src) in hypo-inflammation phase might be beneficial for septic patients. Thus, a precise therapy for sepsis should firstly discriminate the immune status.

Siglec-G is an immunoglobulin-like lectin molecule that recognizes sialic acid and CD24 (17, 26). By interacting with CD24, Siglec-G suppresses inflammatory responses to DAMP such as heat-shock proteins (Hsp70 and Hsp90) and high mobility group box 1 protein (HMGB1) and also regulating TLR activation (23, 24). Siglec-G/10 plays an important role in self–non-self discrimination of innate immunity. In animals, sialic acid-bearing glycans, which are expressed on the cell surface as components of membrane glycoproteins or of a class of glycolipids called gangliosides, forms a key component of the glycocalyx and is critically involved in cell–cell interactions and cell signaling (17, 26). Diverse functions of inflammatory responses induced by DAMP or PAMP may result from different expression levels of Siglec-G and sialic acid-bearing glycans on the membrane, which leads to activation of SHP1 or SHP2, with varying degrees. Furthermore, the interaction of Siglec-G with sialic acid from membrane proteins (like CD24) or other proteins may be disrupted by sialidases such as neuraminidase. Neuraminidase was upregulated in our previous gene expression assay (25). Neuraminidase inhibitor is used as an antiviral drug, typically for influenza infection. Our unpublished data suggested that neuraminidase inhibitor might have a function similar to that of Siglec-G on TLR4-induced signaling and response, indicating that neuraminidase inhibitor might increase the following bacteria-induced inflammation. The function of the neuraminidase inhibitor and *Siglecg* deficiency effect on TLR signaling and responses indicated that Siglec-G was an important member of Siglecs on macrophages. Further research of neuraminidases on inflammation expressed from macrophages, other cells, or pathogens will provide a novel mechanism of innate immunity.

Our previous work showed that Siglec-G associated with c-Cbl caused degradation of RIG-I and reduced production of type I IFN in response to RNA virus infection in a CD24-independent manner (25). Here, we found that Siglec-G promoted TLR-triggered pro-inflammatory cytokines expression both *in vivo* and *in vitro* by regulating Src activation through SHP1. Siglecs can bind sialic acid-modified substrates through immunoglobulin-like receptors, which discriminate between self and non-self and regulate the function of cells in the innate and adaptive immune systems (18, 26). Siglec-G was reported to bind sialic acid on CD24 to downregulate TLR4 signaling in-cis manner (on the same cell) (23, 24). Siglec-G could also participate in autoimmune disease by cooperating with inhibitory receptor FcγRIIb (35). However, FcγRIIb has also a negative function on TLR4 signaling via the ITAM domain (12, 13). Recently, tumor cells that expressed CD24 will inhibit phagocytosis by macrophages through binding Siglec-G in-trans manner (36), which also indicates that different substrate recognition patterns of Siglec-G. We speculated that the interaction with different sialic acid-modified substrates and different expression statuses of Siglec-G upon LPS stimulation might induce different signaling pathways. Different gene backgrounds of control mice, knockout efficiency, and experimental model will also influence the function of Siglec-G on inflammation (23, 24). We focused on function of Siglec-G on the both acute and immunosuppressive phases of LPS-induced sepsis model, which is much different from the cecal ligation and puncture (CLP)-induced sepsis model. The rigid control mice with the same gene background (*Siglecg*^+/−^ vs. *Siglecg*^−/−^), which is more suitable to illustrate the function of Siglec-G. Siglecs are also endocytic receptors that constitutively cycle between the cell surface and intracellular endosome ligand-bearing cargo into the cell, in which the molecular basis for sialic acid specificity remains to be revealed (26). Our lab previously found that Siglec-G negatively regulated cross-presentation in DCs through SHP1. We recently found that Src-LAPF-Cav1 promoted both bacteria and TLR4 endocytosis (37), which indicated that activating Src might increase anti-bacterial innate immunity. Further studies on Siglec-G in different cells by regulating SHP1/2 and Src activation may reveal the molecular mechanism of innate immune responses.

In summary, we reported that Siglec-G-induced Src signaling could orchestrate both inflammatory and anti-inflammatory cytokines by recruiting SHP1 and regulating Src activation (Supplementary Figure 5). Consistent with previous studies that revealed the negative function of Src-Syk signaling on TLR signaling, targeting Src or Siglec-G activation may be promising to treat hyper-inflammatory and hypo-inflammatory responses in sepsis.

## Materials and Methods

### Mice

Mice homozygous for Siglec-G-deficient mice on a C57BL/6J background were generated as described previously (25) and bred in pathogen-free conditions. *Siglecg*^−/−^ heterozygote littermates were derived from the first filial generation mice of *Siglecg*^−/−^ mice mating with WT C57BL/6J mice. Six to eight weeks of age littermate mice was used in the experiments (body weight and sexuality balanced). *Siglecg*^+/−^ and *Siglecg*^−/−^ mice were derived from the *Siglecg*^+/−^ heterozygote littermates. All animal experiments were performed in accordance with the National Institutes of Health Guide for the Care and Use of Laboratory Animals, with the approval of the Scientific Investigation Board of Second Military Medical University, Shanghai.

### Reagents

LPS (O111:B4) used was described previously (11, 25). PP1 was from Calbiochem (San Diego, CA). Antibodies specific to HA-tag, myc-tag, Flag-tag [horseradish peroxidase [HRP] conjugated], CD11b (EPR1344), and the agaroses used in immunoprecipitations were from Abcam Inc. Abs specific for actin, Src, Siglec-G, p65, c-jun, HIF1α and GAPDH, phospho-specific Abs against Src (Tyr416), GSK-3β (Ser9), STAT3 (Tyr705), p65 (Ser536), P38 (Thr180/Tyr182), ERK (Thr202/Tyr204), IKKα/β (Ser176/180), IKBα (Ser32), and c-jun (Ser73) were from Cell Signaling Technology. HRP-conjugated second antibody (TrueBlot) was from eBioscience. Chemical inhibitor PP1 (5 μM) and zanamivir (50 μM) were from Selleck company.

### Plasmid Constructs and Transfection

The recombinant vectors encoding Src (NM_009271), SHP1 (NM_011202.3), HIF1α (NM_001313919), STAT3 (NM_213659), and Siglec-G (NM_172900.3) were constructed by PCR-based amplification from RAW264.7 cDNA and then subcloned into the pcDNA3.1 eukaryotic expression vector (Invitrogen, San Diego, CA) as described previously (11, 25). The CA-Src and Siglec-G mutants were constructed as described previously (11, 25). Transient transfection of plasmids into HEK293T or RAW264.7 cells with jetPEI reagents (Polyplus-transfection Company) was performed following the instructions.

### Cell Culture and ELISA Assays

RAW 264.7 and HEK293T cell lines were obtained from the American Type Culture Collection. Six-week-old mice were injected with 3% (w/v) Merck thioglycollate medium into the peritoneal cavity of each mouse. Three days after the injection, the peritoneal macrophages were collected by flushing the peritoneal cavity with Roswell Park Memorial Institute (RPMI) 1640 and cultured for 1 h and washed with DMEM to clear the non-adherent cells; then the macrophages were further cultured in endotoxin-free DMEM with 10% fetal bovine serum (FBS) (Invitrogen) as previously reported (11, 25). Overexpression of Siglec-G in RAW264.7 cells was transfected with jetPEI (Polyplus) and selected by neomycin (Sigma-Aldrich) as described previously (11). After the stimulation (100 ng/ml of LPS), the concentrations of cytokines in the culture supernatants were determined by ELISA kits (R&D Systems, Minneapolis, MN) as described previously (11, 25).

### RNA Quantification

Quantitative real-time RT-PCR analysis was performed by LightCycler (Roche, Basel, Switzerland) and SYBR RT-PCR kit (Takara, Dalian, China) as described previously (11, 25). Data were normalized to GAPDH expression.

### Immunoprecipitation and Immunoblot

Cells were lysed with radioimmunoprecipitation assay (RIPA) buffer (Cell Signaling Technology, Beverly, MA) or M-PER Protein Extraction Reagent (Pierce, Rockford, IL) supplemented with protease inhibitor cocktail. Protein concentrations of the extracts were measured with bicinchoninic acid (BCA) assay (Pierce). The immunoprecipitation and immunoblot assays were performed as described previously (11, 25).

### Statistical Analysis

Results are given as means plus or minus standard deviation (SD). Comparisons between two groups were performed using Student's *t* test. Statistical significance was determined as *P*-values of <0.05 or 0.01.

## Data Availability Statement

Publicly available datasets were analyzed in this study. This data can be found here: GSE39620. Other raw data supporting the conclusions of this manuscript will be made available by the authors, without undue reservation, to any qualified researcher.

## Ethics Statement

The animal study was reviewed and approved by Scientific Investigation Board of Second Military Medical University, Shanghai.

## Author Contributions

YLu, HY, and CH designed and supervised the study. WL, YLi, KQ, BD, and TL performed the experiments. WL, HY, CH, and YLu analyzed the data and wrote the paper.

### Conflict of Interest

The authors declare that the research was conducted in the absence of any commercial or financial relationships that could be construed as a potential conflict of interest.

## References

[1] Bomans K Schenz J Sztwiertnia I Schaack D Weigand MA Uhle F. Sepsis induces a long-lasting state of trained immunity in bone marrow monocytes. Front Immunol. (2018) 9:2685. 10.3389/fimmu.2018.02685 30510555 PMC6254543

[2] Lin GL McGinley JP Drysdale SB Pollard AJ. Epidemiology and immune pathogenesis of viral sepsis. Front Immunol. (2018) 9:2147. 10.3389/fimmu.2018.02147 30319615 PMC6170629

[3] Peters van Ton M Kox M Abdo WF Pickkers P. Precision immunotherapy for sepsis. Front Immunol. (2018) 9:1926. 10.3389/fimmu.2018.01926 30233566 PMC6133985

[4] Sjaastad FV Condotta SA Kotov JA Pape KA Dail C Danahy DB. Polymicrobial sepsis chronic immunoparalysis is defined by diminished Ag-specific T cell-dependent B cell responses. Front Immunol. (2018) 9:2532. 10.3389/fimmu.2018.02532 30429857 PMC6220049

[5] Talisa VB Yende S Seymour CW Angus DC. Arguing for adaptive clinical trials in sepsis. Front Immunol. (2018) 9:1502. 10.3389/fimmu.2018.01502 30002660 PMC6031704

[6] Shankar-Hari M Phillips GS Levy ML Seymour CW Liu VX Deutschman CS. Developing a new definition and assessing new clinical criteria for septic shock: for the third international consensus definitions for sepsis and septic shock (Sepsis-3). JAMA. (2016) 315:775–87. 10.1001/jama.2016.0289 26903336 PMC4910392

[7] Singer M Deutschman CS Seymour CW Shankar-Hari M Annane D Bauer M The third international consensus definitions for sepsis and septic shock (Sepsis-3). JAMA. (2016) 315:801–10. 10.1001/jama.2016.0287 26903338 PMC4968574

[8] Kotas ME Medzhitov R. Homeostasis, inflammation, and disease susceptibility. Cell. (2015) 160:816–27. 10.1016/j.cell.2015.02.010 25723161 PMC4369762

[9] Liu J Cao X. Cellular and molecular regulation of innate inflammatory responses. Cell Mol Immunol. (2016) 13:711–21. 10.1038/cmi.2016.58 27818489 PMC5101451

[10] Cao X. Self-regulation and cross-regulation of pattern-recognition receptor signalling in health and disease. Nat Rev Immunol. (2016) 16:35–50. 10.1038/nri.2015.8 26711677

[11] Han C Jin J Xu S Liu H Li N Cao X. Integrin CD11b negatively regulates TLR-triggered inflammatory responses by activating Syk and promoting degradation of MyD88 and TRIF via Cbl-b. Nat Immunol. (2010) 11:734–42. 10.1038/ni.1908 20639876

[12] Ivashkiv LB. How ITAMs inhibit signaling. Sci Signal. (2011) 4:pe20. 10.1126/scisignal.2001917 21505184 PMC3261782

[13] Abram CL Lowell CA. The expanding role for ITAM-based signaling pathways in immune cells. Sci STKE. (2007) 2007:re2. 10.1126/stke.3772007re2 17356173

[14] Hu X Han C Jin J Qin K Zhang H Li T. Integrin CD11b attenuates colitis by strengthening Src-Akt pathway to polarize anti-inflammatory IL-10 expression. Sci Rep. (2016) 6:26252. 10.1038/srep26252 27188220 PMC4870583

[15] Goncalves-de-Albuquerque CF Rohwedder I Silva AR Ferreira AS Kurz A. R. M. Cougoule C. The Yin and Yang of tyrosine kinase inhibition during experimental polymicrobial sepsis. Front Immunol. (2018) 9:901. 10.3389/fimmu.2018.00901 29760707 PMC5936983

[16] Fraschilla I Pillai S. Viewing Siglecs through the lens of tumor immunology. Immunol Rev. (2017) 276:178–91. 10.1111/imr.12526 28258691 PMC5860639

[17] Mahajan VS Pillai S. Sialic acids and autoimmune disease. Immunol Rev. (2016) 269:145–61. 10.1111/imr.12344 26683151 PMC4769436

[18] Pillai S Netravali IA Cariappa A Mattoo H. Siglecs and immune regulation. Annu Rev Immunol. (2012) 30:357–92. 10.1146/annurev-immunol-020711-075018 22224769 PMC3781015

[19] Crocker PR Paulson JC Varki A. Siglecs and their roles in the immune system. Nat Rev Immunol. (2007) 7:255–66. 10.1038/nri2056 17380156

[20] Wang J Sun J Liu LN Flies DB Nie X Toki M. Siglec-15 as an immune suppressor and potential target for normalization cancer immunotherapy. Nat Med. (2019) 25:656–66. 10.1038/s41591-019-0374-x 30833750 PMC7175920

[21] Li N Zhang W Wan T Zhang J Chen T Yu Y. Cloning and characterization of Siglec-10, a novel sialic acid binding member of the Ig superfamily, from human dendritic cells. J Biol Chem. (2001) 276:28106–12. 10.1074/jbc.M100467200 11358961

[22] Ding Y Guo Z Liu Y Li X Zhang Q Xu X. The lectin Siglec-G inhibits dendritic cell cross-presentation by impairing MHC class I-peptide complex formation. Nat Immunol. (2016) 17:1167–75. 10.1038/ni.3535 27548433

[23] Chen GY Chen X King S Cavassani KA Cheng J Zheng X. Amelioration of sepsis by inhibiting sialidase-mediated disruption of the CD24-SiglecG interaction. Nat Biotechnol. (2011) 29:428–35. 10.1038/nbt.1846 21478876 PMC4090080

[24] Chen GY Tang J Zheng P Liu Y. CD24 and Siglec-10 selectively repress tissue damage-induced immune responses. Science. (2009) 323:1722–5. 10.1126/science.1168988 19264983 PMC2765686

[25] Chen W Han C Xie B Hu X Yu Q Shi L. Induction of Siglec-G by RNA viruses inhibits the innate immune response by promoting RIG-I degradation. Cell. (2013) 152:467–78. 10.1016/j.cell.2013.01.011 23374343

[26] Bordon Y. Inflammation: Live long and prosper with Siglecs. Nat Rev Immunol. (2015) 15:266–7. 10.1038/nri3851 25882243

[27] Gruber S Hendrikx T Tsiantoulas D Ozsvar-Kozma M Goderle L Mallat Z. Sialic acid-binding immunoglobulin-like lectin G promotes atherosclerosis and liver inflammation by suppressing the protective functions of B-1 cells. Cell Rep. (2016) 14:2348–61. 10.1016/j.celrep.2016.02.027 26947073 PMC4802221

[28] Ivashkiv LB. A signal-switch hypothesis for cross-regulation of cytokine and TLR signalling pathways. Nat Rev Immunol. (2008) 8:816–22. 10.1038/nri2396 18787561 PMC2581615

[29] Qin K Han C Zhang H Li T Li N Cao X. NAD(+) dependent deacetylase Sirtuin 5 rescues the innate inflammatory response of endotoxin tolerant macrophages by promoting acetylation of p65. J Autoimmun. (2017) 81:120–9. 10.1016/j.jaut.2017.04.006 28461090

[30] Ouyang W Rutz S Crellin NK Valdez PA Hymowitz SG. Regulation and functions of the IL-10 family of cytokines in inflammation and disease. Annu Rev Immunol. (2011) 29:71–109. 10.1146/annurev-immunol-031210-101312 21166540

[31] An H Hou J Zhou J Zhao W Xu H Zheng Y. Phosphatase SHP-1 promotes TLR- and RIG-I-activated production of type I interferon by inhibiting the kinase IRAK1. Nat Immunol. (2008) 9:542–50. 10.1038/ni.1604 18391954

[32] Gilroy DW Yona S. HIF1alpha allows monocytes to take a breather during sepsis. Immunity. (2015) 42:397–9. 10.1016/j.immuni.2015.02.016 25786169

[33] Zhu J Luo L Tian L Yin S Ma X Cheng S. Aryl hydrocarbon receptor promotes IL-10 expression in inflammatory macrophages through Src-STAT3 signaling pathway. Front Immunol. (2018) 9:2033. 10.3389/fimmu.2018.02033 30283437 PMC6156150

[34] Horiguchi H Loftus TJ Hawkins RB Raymond SL Stortz JA Hollen MK. Innate immunity in the persistent inflammation, immunosuppression, and catabolism syndrome and its implications for therapy. Front Immunol. (2018) 9:595. 10.3389/fimmu.2018.00595 29670613 PMC5893931

[35] Muller J Lunz B Schwab I Acs A Nimmerjahn F Daniel C. Siglec-G deficiency leads to autoimmunity in aging C57BL/6 Mice. J Immunol. (2015) 195:51–60. 10.4049/jimmunol.1403139 25987743

[36] Barkal AA Brewer RE Markovic M Kowarsky M Barkal SA Zaro BW. CD24 signalling through macrophage Siglec-10 is a target for cancer immunotherapy. Nature. (2019) 572:392–6. 10.1038/s41586-019-1456-0 31367043 PMC6697206

[37] Li T Qin K Li N Han C Cao X. An endosomal LAPF is required for macrophage endocytosis and elimination of bacteria. Proc Natl Acad Sci USA. (2019) 116:12958–63. 10.1073/pnas.1903896116 31189603 PMC6601291

