# Supplementary material for: Siglec-G Deficiency Ameliorates Hyper-Inflammation and Immune Collapse in Sepsis via Regulating Src Activation
Source: Front Immunol. 2019 Nov 7;10:2575. doi: 10.3389/fimmu.2019.02575 (PMC6859834; doi:10.3389/fimmu.2019.02575)
Supplement: Supplementary file 1 [file Data_Sheet_1.docx]

Supporting materials for

**Siglec-G Deficiency Ameliorates Hyper-inflammation and Immune Collapse in Sepsis via Regulating Src Activation**

Wenqian Li^1#^, Yinjiao Li^2,#^, Kewei Qin^3#^, Baixiang Du^4#^, Tianliang Li^1^ , Hongbin Yuan^4*^, Chaofeng Han^1*^, Yan Luo^2*^

Supplementary Figures S1-S5

**Supplementary Figure 1.** Q-PCR of Siglec-1, Siglec-E and Siglec-G in peritoneal macrophages (6 × 10^5^) stimulated with LPS (100 ng/ml) for indicated hours. Data are representative of three independent experiments with similar results and presented as means ± SD. ＊P < 0.01.





**Supplementary Figure 2.** (*A)* ELISA of IL-6, TNF-α and IL-10 in supernatant from *Siglecg^+/+^ , Siglecg^+/-^* and *Siglecg*^−/−^ peritoneal macrophages (6 × 10^5^) stimulated with LPS (100 ng/ml) for 6h or 12h. Data are representative of three independent experiments with similar results and presented as means ± SD. # P >0.05,＊P < 0.01.


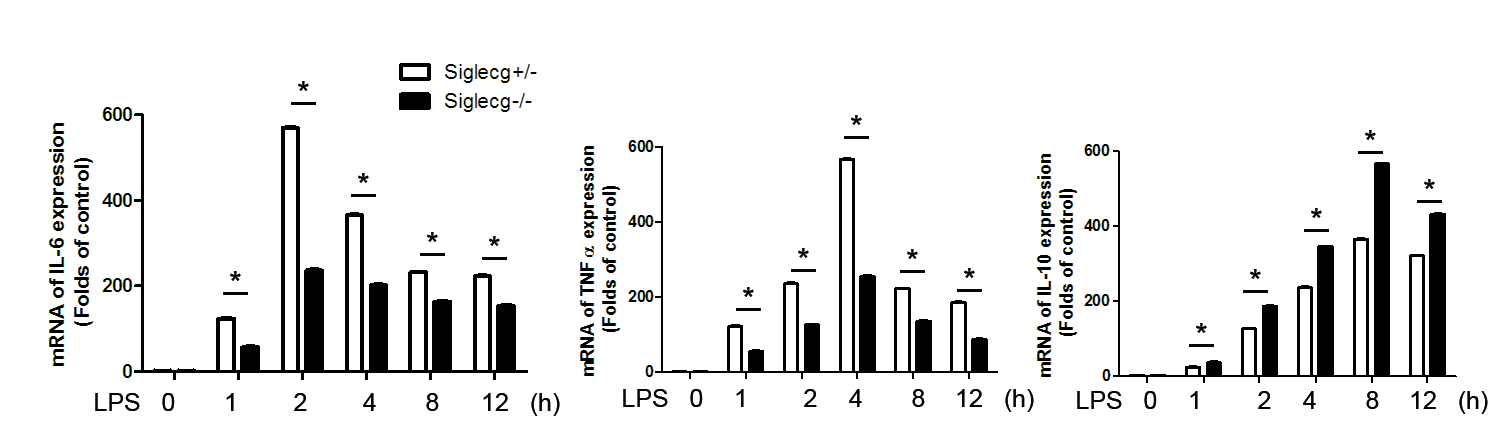


**Supplementary Figure 3.** Q-PCR of of IL-6, TNF-α and IL-10 of *Siglecg*^+/-^ and *Siglecg* ^−/−^ peritoneal macrophages (6 × 10^5^) stimulated with LPS (100 ng/ml) for indicated hours. Data are representative of three independent experiments with similar results and presented as means ± SD.＊P < 0.01.





**Supplementary Figure 4.** Q-PCR of of IL-6, TNF-α and IL-10 of *Siglecg*^+/-^ and *Siglecg* ^−/−^ peritoneal macrophages (6 × 10^5^) pretreated with DMSO or PP1 for 30 minutes then stimulated with LPS (100 ng/ml) for 4 hours. Data are representative of three independent experiments with similar results and presented as means ± SD.＊P < 0.01.


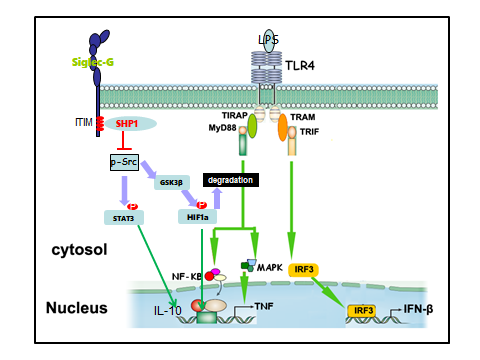


**Supplementary Figure 5.** Siglec-G orchestrates inflammation by regulating HIF1α and STAT3 activation through SHP1-Src.
